# Adipocyte-specific deletion of PIP5K1c reduces diet-induced obesity and insulin resistance by increasing energy expenditure

**DOI:** 10.1186/s12944-021-01616-4

**Published:** 2022-01-07

**Authors:** Guan Huang, Cuishan Yang, Sheng Guo, Miaoling Huang, Liping Deng, Ying Huang, Puxin Chen, Feng Chen, Xiaohong Huang

**Affiliations:** 1 Department of Pathology, Shenzhen Clinical Medical College, Guangzhou University of Chinese Medicine, Longgang District Central Hospital of Shenzhen, Shenzhen, 518116 Guangdong China; 2 Department of Nursing, Shenzhen Clinical Medical College, Guangzhou University of Chinese Medicine, Longgang District Central Hospital of Shenzhen, Shenzhen, 518116 Guangdong China; 3 Department of Medical Administration, Shenzhen Clinical Medical College, Guangzhou University of Chinese Medicine, Longgang District Central Hospital of Shenzhen, Shenzhen, 518116 Guangdong China; 4 Department of Metabolism and Endocrinology, Shenzhen Clinical Medical College, Guangzhou University of Chinese Medicine, Longgang District Central Hospital of Shenzhen, Shenzhen, 518116 Guangdong China; 5 Department of Plastic Surgy, Shenzhen Clinical Medical College, Guangzhou University of Chinese Medicine; Longgang District Central Hospital of Shenzhen, Shenzhen, 518116 Guangdong China

**Keywords:** Obesity, PIP5K1c, Adipose tissue, Energy expenditure, Adipogenesis

## Abstract

**Background:**

Phosphatidylinositol 4-phosphate 5-kinase type I c (PIP5K1c) catalyses the synthesis of phospholipid phosphatidylinositol 4,5-bisphosphate (PIP2) by phosphorylating phosphatidylinositol 4 phosphate, which plays multiple roles in regulating focal adhesion formation, invasion, and cell migration signal transduction cascades. Here, a new physiological mechanism of PIP5K1c in adipocytes and systemic metabolism is reported.

**Methods:**

Adipose-specific conditional knockout mice were generated to delete the PIP5K1c gene in adipocytes. In addition, in vitro research investigated the effect of PIP5K1c deletion on adipogenesis.

**Results:**

Deletion of PIP5K1c in adipocytes significantly alleviated high fat diet (HFD)-induced obesity, hyperlipidaemia, hepatic steatosis, and insulin resistance. PIP5K1c deficiency in adipocytes also decreased adipocyte volume in HFD-induced obese mice, whereas no significant differences were observed in body weight and adipose tissue weight under normal chow diet conditions. PIP5K1c knockout in adipocytes significantly enhanced energy expenditure, which protected mice from HFD-induced weight gain. In addition, adipogenesis was markedly impaired in mouse stromal vascular fraction (SVF) from PIP5K1c-deleted mice.

**Conclusion:**

Under HFD conditions, PIP5K1c regulates adipogenesis and adipose tissue homeostasis. Together, these data indicate that PIP5K1c could be a novel potential target for regulating fat accumulation, which could provide novel insight into the treatment of obesity.

**Supplementary Information:**

The online version contains supplementary material available at 10.1186/s12944-021-01616-4.

## Introduction

Obesity is a serious health concern and is associated with higher risks of developing diabetes, fatty liver and cardiovascular disease [1, 2]. The prevalence of obesity has nearly tripled since 1975 worldwide. There are currently 650 million adults affected by obesity (body mass index [BMI] ≥30 kg/m^2^), and the number is estimated to be greater than 1 billion by 2050 [3]. Adipose tissue plays a crucial role in regulating homoeostasis, and the expression of inflammatory cytokines in adipose tissue correlates with BMI and insulin resistance [4–7]. Adipose tissue includes white adipose tissue (WAT) and brown adipose tissue (BAT). WAT specializes in lipid storage and adipokine secretion to regulate energy storage and systemic insulin sensitivity [8]. BAT’s function is for energy dissipation and heat generation [9]. In addition, upon cold exposure and exercise, WAT can be switched into beige adipocytes in a process termed beiging [10, 11], the functions and cellular features of which are similar to those of BAT.

Phosphorylated derivatives of phosphatidylinositol play critical roles in the lipid regulation of many physiological processes in eukaryotic cells [12]; e.g., phosphatidylinositol 4,5-bisphosphate (PIP2), primarily synthesized by type I phosphatidylinositol 4-phosphate 5-kinase (PIP5K1) catalysing the phosphorylation of phosphatidylinositol 4-phosphate [13, 14], is widely known to produce lipid second messengers. To date, three isoforms of PIP5K1 have been identified that are able to synthesize PIP2. The three isoforms of PIP5K1 are heterogeneous in location, structure, and expression level in different tissues. PIP5K1a is mainly localized in membrane ruffles [15], PIP5K1b is found at endosomes [16], and PIP5KIc is found at nerve terminals by targeting focal adhesions (FAs) [17–19]. Evidence suggests that the production of PIP2 on the membrane associated with focal adhesions involves PIP5K1c [20–22]. During FA dynamics, the interaction of talin and PIP5K1c must be tightly controlled. PIP5K1c knockout mice showed early lethality accompanied by defects in the development of different organs [23, 24]. Previous studies also showed that focal adhesion proteins were important for adipocyte homeostasis and survival [25, 26]. These reports suggest that focal adhesion proteins are mediators of metabolic signalling and that PIP5K1c is essential for FA dynamics to control adipocyte homeostasis. However, there is no direct evidence to report the role of PIP5K1c in adipocytes to regulate adipose tissue homeostasis and obesity-related diseases.

In the present study, the authors explored the function of PIP5K1c in adipose tissue development and homeostasis using a transgenic mouse model in vivo. The authors found that deletion of PIP5K1c in adipocytes reduced adipose tissue mass under HFD conditions and ameliorated insulin resistance. In vitro, loss of function of PIP5K1c results in impaired adipogenesis. These findings probably provide new insight into the role of PIP5K1c in obesity and obesity-related diseases.

## Materials and methods

### Mice, diet and treatments

Mice with adipocyte-specific deletion of PIP5K1c were generated using the Cre-loxP system and crossing PIP5K1c flox mice (Shanghai Model Organism, Shanghai, China) with *adiponectin*-cre mice (Jackson Labs, Bar Harbour, ME). Mice were bred and maintained under a light/dark cycle (12 h/12 h) with standard food and water ad libitum. For the HFD study, 6-week-old mice were fed an HFD diet (#12492, research diet, NJ, USA) for 16 weeks. All mice and procedures were performed on a protocol approved by the Institutional Animal Care and Use Committee of Guangzhou University of Chinese Medicine (JY2020046).

### qRT–PCR

Total RNA was extracted from adipose tissue using TRIzol Reagent according to previously described methods [27]. Synthesis of complementary DNA was performed using a cDNA Synthesis Kit (Applied Biosystems, Shanghai, China). Quantitative reverse transcription polymerase chain reaction (qRT–PCR) was performed with SYBR Green Master Mix (Bio–Rad, Shanghai, China) in a 96-well plate using a PCR system (Bio–Rad, Shanghai, China). The ΔΔCt method was used to determine relative gene expression using Gapdh as a reference gene. The primer sequences are presented in Supplementary Table 1.

### Western blotting

Total proteins from tissue and cell samples were lysed in RIPA buffer with protease inhibitor as described previously [28]. In brief, adipose tissue was cut into small pieces in RIPA buffer (20 mmol/L Tris-HCl (pH 8.0), 150 mmol/L NaCl, 0.1% SDS, 0.5% NP-40, 1 mmol/L EDTA and a protease inhibitor cocktail), homogenized for 20 mins, and centrifuged at 13,000 rpm at 4 °C for 10 min. After the protein concentration was quantified using a BCA assay kit, 30 μg of protein was separated by 10% SDS–PAGE and transferred to a PVDF membrane. After blocking for 1 h at room temperature, the membranes were incubated with primary antibodies at 4 °C overnight. Blots were subsequently washed and then incubated with secondary antibodies. Signals were developed using an ECL kit (Bio–Rad, Shanghai, China) and quantified in ImageJ software. Antibody information is listed in Supplementary Table 2.

### Stromal vascular fraction (SVF) isolation and differentiation

The SVF was extracted from subWAT using methods that were described previously [26]. In brief, the adipose tissue was cut into pieces, digested for 45 mins with collagenase D in a water bath at 37 °C and filtered through a 70-μm filter after terminating digestion. The cells were centrifuged at 1500 rpm for 10 min and then cultured in medium containing DMEM/F12 with 10% FBS (grown medium, GM) at 37 °C with 5% CO_2_. After reached 100% confluence, the preadipocytes were cultured with DMEM/F12 supplemented with 0.5 mM isobutyl methylxanthine (IBMX), 1 μM dexamethasone and 5 μg/ml insulin for 48 h, followed by GM and 5 μg/ml insulin every two days.

### Histology analysis

Adipose and liver tissues were fixed and processed for paraffin embedding, cut into 5-μm sections and stained using haematoxylin and eosin (H&E). Adipocyte areas were quantified manually using ImageJ software.

### Metabolic cage

O_2_/CO_2_ consumption, heat production and respiratory exchange ratio (RER) were detected using a Columbus Oxymax/CLAMS system (Columbus Instrument, Columbus, OH, USA). Mice were monitored individually for 24 h, and data were collected at a time interval of 30 min.

### Glucose and insulin tolerance tests and measurements of triglycerides, cholesterol and NEFAs

Glucose tolerance test (GTT) and insulin tolerance test (ITT) were performed as described previously [26]. For GTT, mice were fasted for 16 h followed by intraperitoneal injection of 2 g/kg as previously described. For ITT, mice were fasted for 4 h followed by intraperitoneal injection of insulin at 0.75 U/kg body weight. Blood glucose concentration was measured by tail bleed at the indicated time point. Serum levels of triglycerides (TG), total cholesterol (TCH) and nonestesterified fatty acid (NEFA) were measured using triglyceride (Jiancheng, Nanjing, China), total cholesterol (Jiancheng, Nanjing, China), and mouse NEFA (Jiancheng, Nanjing, China) kits.

### Statistical analyses

Data were shown as means (SEM). For two groups, significance was analysed using an unpaired, 2-tailed Student’s t test, and a value of *P* < 0.05 was considered statistically significant.

## Results

### Generation of adipose tissue PIP5K1c knockout mice

**Fig. 1:**
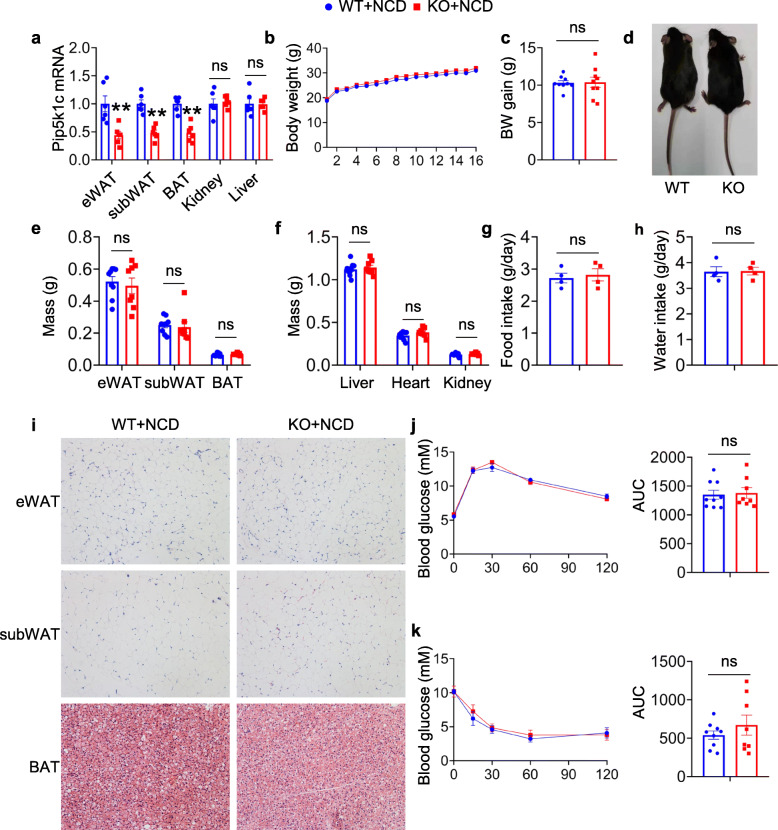
Generation of adipocyte-specific PIP5K1c knockout mice. **a** qPCR analysis of PIP5K1c mRNA expression in different organs of 20-week-old WT and KO mice. **b** Body weight and **c** body weight gain of WT and KO mice fed with NCD for 20 weeks, *N* = 8–9/group. **d** Morphology of WT and KO mice fed with NCD. **e** Adipose tissue mass including eWAT, subWAT and BAT after sacrifice of mice, *N* = 8–9/group. **f** Organ weight of liver, heart and kidney after sacrifice of mice, *N* = 8–9/group. **g** Food intake and **h** water intake statistics of WT and KO mice fed with NCD. **i** Representative image of H&E staining of adipose tissue from WT and KO mice with NCD diet, Scale bar: 50 μm. (**j, k**) Glucose tolerance test **j** and Insulin tolerance test **k** in WT and KO mice fed an NCD, Right panel, area under the curve, *N* = 8–9/group. *P < 0.05; **P < 0.01

In the present study, the authors used PIP5K1c-floxed mice crossed with adiponectin-Cre transgenic mice to investigate the metabolic effect induced by PIP5K1c deletion in adipocytes, hereafter named KO. The levels of PIP5K1c in subcutaneous white adipose tissue (subWAT), epididymal white adipose tissue (eWAT) and BAT were markedly reduced in KO mice in comparison with flox/flox (WT) mice (Fig. 1a). There was no decrease in PIP5K1c in nonadipose tissue from KO mice (Fig. 1a). The authors monitored the body weights of the two genotypes of mice from 6 weeks old until they reached age 22 weeks. During this period, KO mice did not display significant differences in body weight or body weight gain when fed a normal chow diet (NCD) (Fig. 1b-d). Until 22 weeks of age, when the two groups of mice were sacrificed, no significant difference was observed in adipose tissue weight between WT and KO mice (Fig. 1e). Other nonadipose tissue weights, such as heart, kidney and liver, were similar to those of the littermate controls (Fig. 1f). Furthermore, no difference in food or water intake was observed between these two groups of mice (Fig. 1g, h). The morphologies of eWAT, subWAT adipocytes and BAT adipocytes between the control and KO mice were similar under NCD challenge by H&E staining (Fig. 1i). Concomitantly, the authors also assessed glucose homeostasis. There was no difference in glucose tolerance test (Fig. 1j) or insulin tolerance test between control mice and KO mice fed a normal chow diet (Fig. 1k).

### PIP5K1c knockout in adipocytes ameliorates HFD-induced obesity

**Fig. 2:**
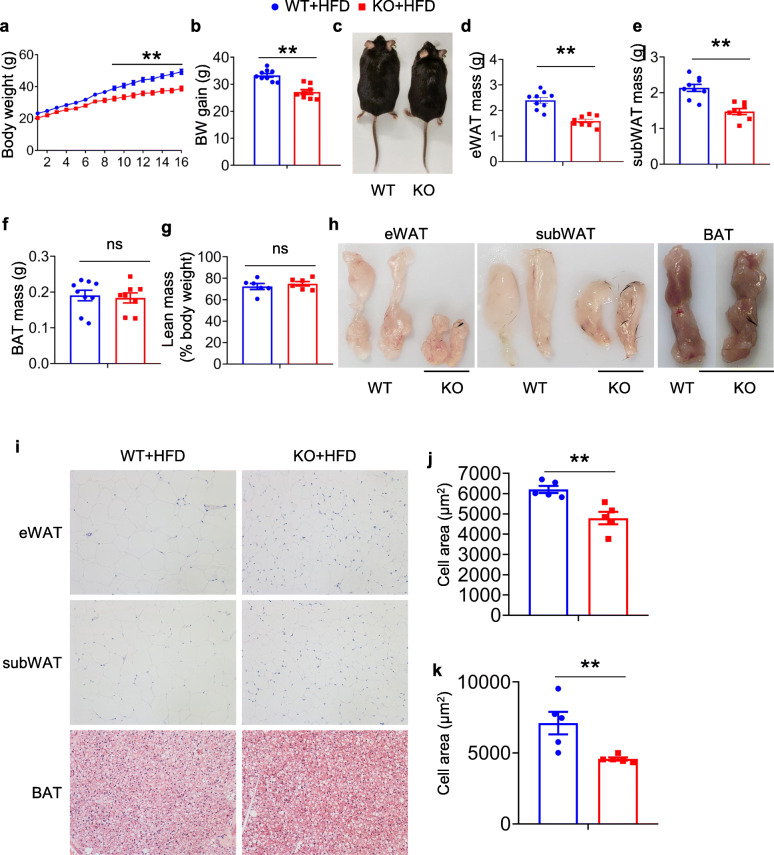
PIP5K1c KO mice are resistant to HFD induced obesity. (**a-c**) Growth curves **a**, body weight gain **b** and body shape **c** of HFD-fed WT or KO mice. **d** eWAT mass of WT and KO mice after 16 weeks HFD feeding, *N* = 8–9/group. **e** subWAT mass of WT and KO mice after 16 weeks HFD feeding, *N* = 8–9/group. **f** BAT mass of WT and KO mice after 16 weeks HFD feeding, *N* = 8–9/group. **g** Lean mass of WT and KO mice after 16 weeks HFD feeding, *N* = 6. **h** Representative pictures of different adipose tissue from WT and KO mice after 16 weeks HFD feeding. **i** H&E staining of different adipose tissues in WT and KO mice after 16 weeks HFD feeding. **j** Adipocyte average size in eWAT of HFD-fed WT and KO mice, *N* = 5/group. **k** Adipocyte average size in subWAT of HFD-fed WT and KO mice, *N* = 5/group. *P < 0.05; **P < 0.01

To determine the roles of PIP5K1c in HFD-induced obesity, beginning at 6 weeks of age, KO mice and control mice were fed a HFD for 16 weeks. The body weight and body weight gain were less than those of WT mice (Fig. 2a-c). KO mice gained less WAT mass than WT mice (Fig. 2d-h). However, BAT and lean mass was similar in KO and WT mice (Fig. 2f, g). The results of the histology analysis showed significant reductions in adipocyte diameters in the eWAT and subWAT of KO mice (Fig. 2i-k). Similar results were also observed in the female groups (Supplementary Fig. 1). These data demonstrated that PIP5K1c knockout in adipocytes could mitigate the effect of HFD-induced obesity.

### KO mice fed a HFD present improved metabolic profiles

**Fig. 3:**
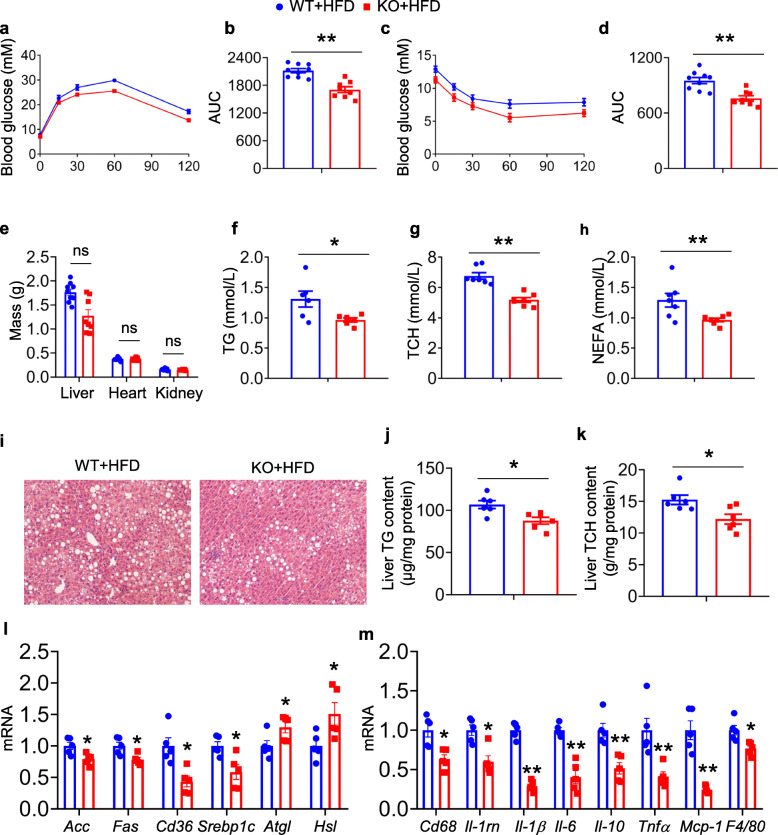
Adipocyte-deficient of PIP5K1c show the improved metabolic profile under HFD feeding condition. **a** Blood glucose concentration during glucose tolerance tests (GTT) in HFD feeding WT and KO mice. **b** Area under curve (AUC) calculated based on panel a, *N* = 8–9/group. **c** Blood glucose concentration during insulin tolerance tests (ITT) in HFD feeding WT and KO mice. **d** Area under curve (AUC) calculated based on panel c, *N* = 8–9/group. **e** Organ mass of WT and KO mice after 16 weeks HFD feeding, *N* = 8–9/group. **f** Plasma triglyceride (TG), **g** total cholesterol (TCH), and **h** nonesterified fatty acid (NEFA) levels of WT and KO mice on HFD for 16 weeks, *N* = 7/group. **i** Representative image of H&E staining of liver from WT and KO mice with 16 weeks HFD diet. **j, k** Liver triglyceride (TG) and total cholesterol (TCH) content in WT and KO mice after 16 weeks HFD feeding, *N* = 6/group. **l** qPCR analysis of mRNA levels of lipogenesis genes in liver from WT and KO mice after 16 weeks HFD feeding. **m** qPCR analysis of mRNA levels of inflammation genes in livers from WT and KO mice after 16 weeks HFD feeding, *N* = 6 per group. *P < 0.05; **P < 0.01

Next, the authors performed GTT and ITT to assess whether the decreased lipid storage affords beneficial effects on homeostasis in KO mice. The decreased area under the curve (AUC) of GTT and ITT (Fig. 3a-d) illustrated that compared with the WT HFD mice, a more prominent clearance of plasma glucose and ameliorated insulin sensibility were observed in KO HFD mice. Furthermore, KO mice showed slightly decreased liver weight, although there was no significant difference in comparison with WT mice fed a HFD (Fig. 3e). In addition, the circulating levels of metabolic profiles, including fasting plasma TG, TCH, and NEFA, were significantly lower in KO mice than in WT mice after 16 weeks of HFD feeding (Fig. 3f-h). H&E staining and decreased liver TG and TCH levels showed substantially lower lipid storage in the livers of KO mice than in WT mice under HFD conditions (Fig. 3i-k). The mRNA levels of sterol regulatory element-binding protein-1c (Srebp-1c) and fatty acid synthesis genes, e.g., fatty acid synthase (FAS) and Acetyl-CoA carboxylase (ACC), in the livers of KO mice were significantly decreased, whereas lipolysis genes, e.g., adipose triglyceride lipase (ATGL) and Hormone-Sensitive Lipase (HSL), were increased (Fig. 3l). In addition, the mRNA levels of inflammatory genes, e.g., F4/80, CD68, monocyte chemoattractant protein 1 (MCP-1) and tumor necrosis factor-α (TNF-α), were downregulated in KO livers (Fig. 3m). Collectively, these findings suggested that loss of adipocyte PIP5K1c ameliorated systemic insulin resistance and HFD-induced liver steatosis.

### PIP5K1c knockout enhances energy expenditure in HFD mice

**Fig. 4:**
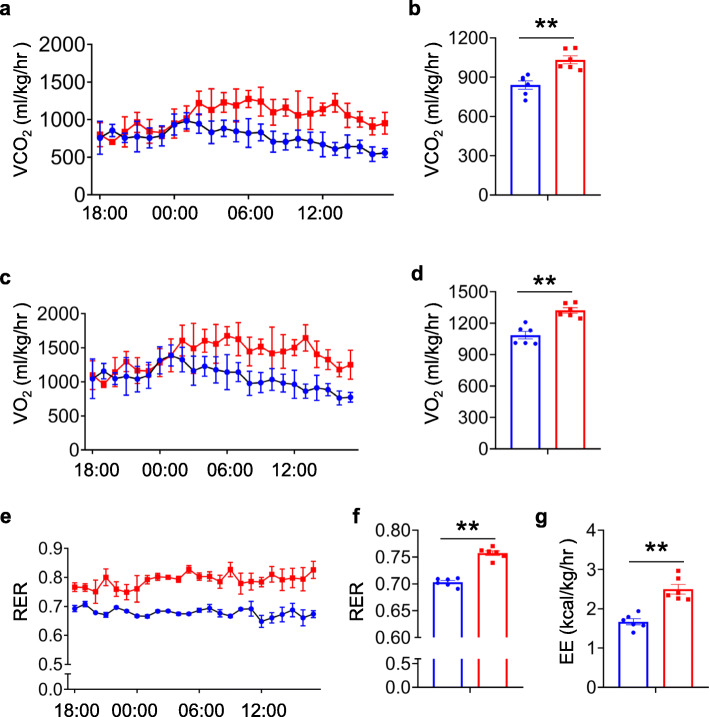
PIP5K1c deficiency enhance energy expenditure in mice fed with HFD. **a, b** Carbon dioxide expiration (VCO2) and **c, d** oxygen consumption (VO2) was determined by metabolic cages in WT and KO mice after HFD feeding, *N* = 6/group. **e, f** Respiratory exchange ratio (RER) over 24 h for WT and KO mice after 16 weeks HFD feeding. **g** Energy expenditure (EE) was estimated by the amount of O2 consumption and CO2 production in WT and KO mice after 16 weeks HFD feeding, *N* = 6/group. *P < 0.05; **P < 0.01

To illustrate the potential mechanism underlying the markedly reduced fat tissue storage in KO mice, the authors used metabolic cages to determine the contribution of PIP5K1c knockout to energy expenditure (EE). Comparing the KO HFD mice with WT controls, CO_2_ production (Fig. 4a, b) and O_2_ consumption (Fig. 4c, d) were significantly increased during both the dark and light phases under HFD feeding. Concomitantly, the respiratory exchange ratio (RER) was increased in KO mice (Fig. 4e, f), suggesting increased utilization of carbohydrates over fatty acids. Moreover, the EE estimated by O_2_ consumption and CO_2_ production was significantly increased in KO HFD mice (Fig. 4g).

### PIP5K1c deficiency impairs adipocyte differentiation

**Fig. 5:**
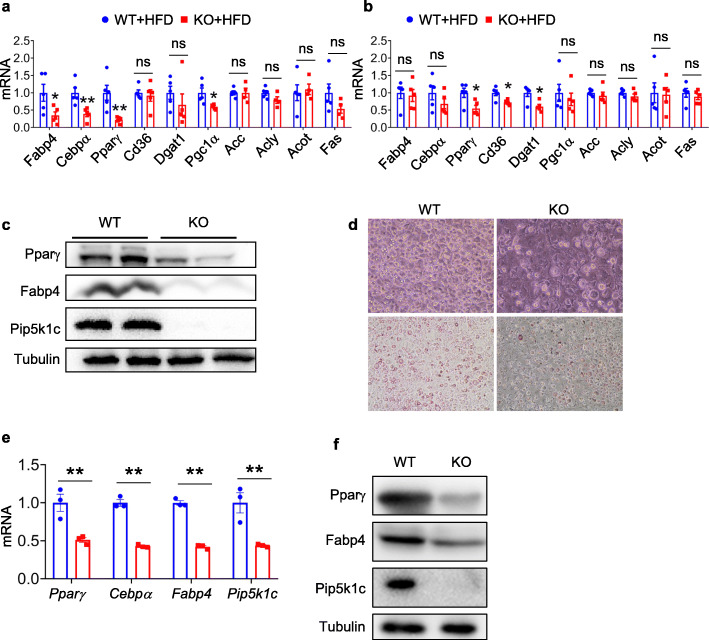
Loss of PIP5K1c impairs adipogenesis. **a, b** qPCR analysis of adipocyte-specific marker genes in adipocytes from eWAT and subWAT of HFD-fed mice (*N* = 5). **c** Western blotting for adipogenesis proteins in adipocytes from eWAT of HFD-fed mice. **d** Oil-Red O staining of WT and KO SVF at day 8 of differentiation. **e** qPCR analysis of adipogenesis genes in adipocytes from WT and KO mice after differentiation. **f** Western blotting for adipogenesis proteins in adipocytes from WT and KO mice SVF after differentiation. *P < 0.05 and **P < 0.01

PIP5K1c deficiency in adipocyte mice decreased adipose tissue mass, especially under HFD challenge, which suggested the potential effect of PIP5K1c on adipocyte differentiation. To investigate the function of PIP5K1c in adipocyte differentiation, the authors examined adipogenesis markers in WAT of WT and KO mice. As shown in Fig. 5a and b, deletion of PIP5K1c in adipocytes significantly decreased adipogenesis genes in eWAT but not subWAT. Consistent with the gene expression data, adipogenesis proteins, such as PPARγ and fabp4, were both decreased in the eWAT of KO mice (Fig. 5c). However, no difference of lipogenesis proteins were observed between WT and KO mice after HFD feeding by western blotting test (Supplementary Fig. 2). Next, the authors used primary adipocytes isolated from WT and KO mice to assess whether loss of PIP5K1c may affect adipogenesis. Oil red O staining showed that deletion of PIP5K1c reduced the number of oil red O adipocytes, indicating impaired adipogenesis (Fig. 5d). The gene and protein expression levels of adipogenic markers also indicated that PIP5K1c deletion in adipocytes impaired adipogenesis (Fig. 5e, f). Furthermore, overexpression of PIP5K1c in 3 T3-L1 cells promoted adipogenesis (Supplementary Fig. 3). These data indicated that PIP5K1c is essential for adipogenesis.

### PIP5K1c regulates adipose remodelling followed by dietary changes

**Fig. 6:**
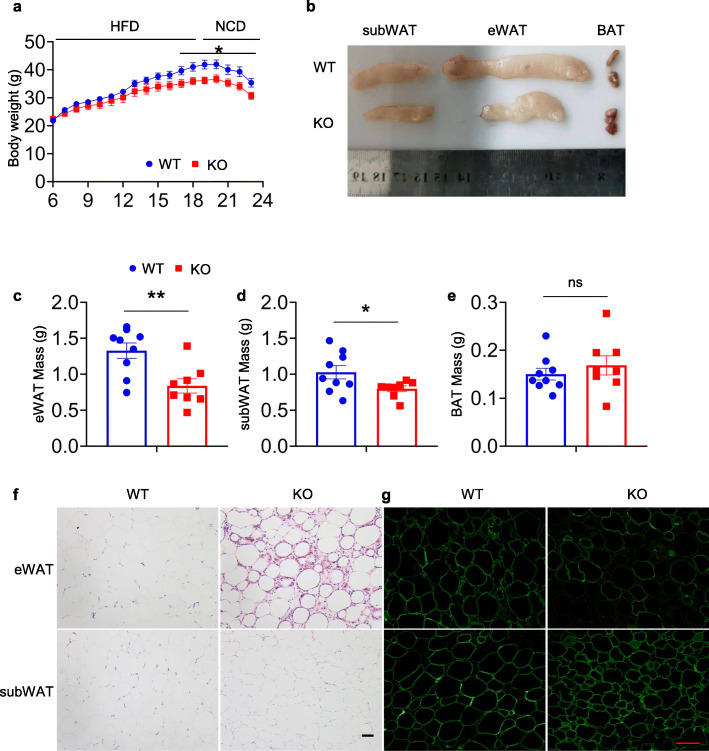
Loss of PIP5K1c in adipocyte results in defects in adipose tissue remodeling in response to HFD/NCD changes. **a** Growth curves of 12 weeks HFD-fed control and KO mice switched to the NCD for another 4 weeks. **b** Representative photographs of adipose tissue. **c-e** eWAT, subWAT and BAT weight from control and KO mice, *N* = 8–9/group. **f** H/E staining of subWAT and eWAT from HFD/NCD-fed male mice. **g** Immunofluorescence staining for perilipin of eWAT from control and KO mice fed HFD/NCD. *P < 0.05 and **P < 0.01

In response to nutritional status, adipose tissue can expand or contract physically. To investigate the role of PIP5K1c in the contraction capacity of adipose tissue after the termination of the HFD, the authors changed the KO and control mice to the NCD for another 4 weeks. Similar reductions in body weight in response to the HFD/NCD change were observed in the two groups (Fig. 6a). Moreover, KO mice had smaller and discoloured eWAT (Fig. 6b-e). Histological examination suggested adipose remodelling, as evidenced by a normal subWAT phenotype but severe degeneration of the integrity of eWAT in PIP5K1c knockout mice (Fig. 6f). Immunofluorescence staining showed that the loss of perilipin in eWAT in KO mice, suggesting increased adipocyte death (Fig. 6g). Taken together, the results showed that PIP5K1c plays a critical role in adipose tissue remodelling in response to dietary changes.

## Discussion

Obesity is a global public health crisis that is associated with high morbidity and mortality. The disease burden of obesity merits more attention from stakeholders and further aggressive intervention. Drugs that are FDA-approved for obesity can be classified into two types – appetite reduction and satiety enhancement – and both work through central nervous system (CNS) pathways. However, undesirable adverse effects including increased in heart rate and blood pressure are often observed after drug administration [29]. In the present study, the results showed that a lack of PIP5K1c in adipocytes significantly alleviated HFD-induced fat accumulation, decreased adipose tissue mass, improved insulin sensitivity and decreased ectopic fatty acid accumulation in the liver, indicating that PIP5K1c is a driving force for diet-induced obesity and metabolic syndrome. These data suggest that PIP5K1c may have the potential to serve as a new target molecule for obesity therapy.

In many cells, including adipocytes, FA is essential for growth and survival and involves phosphoinositol 3-kinase (PI3K)/AKT activity modulated by FAK [30, 31]. Previous studies have shown that PIP5K1c is associated with talin and proved that PIP5K1c contributes to the formation of focal adhesion [32–35]. Adherence to the extracellular matrix stimulates the generation of PIP2 and potentially modulates focal adhesion assembly around clusters of integrins [36]. Anderson et al. [37] demonstrated that PIP5K1c is associated with N-cadherin and can regulate its trafficking, which is critical for the formation of adherens junctions. These findings suggest that deletion of PIP5K1c in adipocytes ameliorates HFD-induced adiposity expansion and insulin resistance.

The results of the present study identify a novel role of adipocyte PIP5K1c signalling in the control of glucose metabolism. The authors demonstrate that PIP5K1c deletion does not markedly impact glucose metabolism in mice fed NCD. In contrast, and of particular interest, ablation of PIP5K1c in adipocytes in mice fed a HFD ameliorates metabolic abnormalities, including decreased fasting blood glucose levels, improved insulin sensitivity, ameliorated glucose intolerance and ameliorated fatty liver. These results suggest that altered PIP5K1c expression in adipocytes may play a role in the pathogenesis of certain metabolic diseases, such as type 2 diabetes, obesity, fatty liver and metabolic syndrome in humans. Thus, the findings of the present study highlight the importance of investigating the role of adipocyte PIP5K1c signalling in human diseases.

In the present study, PIP5K1c expression in adipose tissue did not appear to regulate adiposity and glucose metabolism under NCD, and the differences were probably too small to result in changes in total body metabolism. Improved glucose homeostasis was observed under HFD conditions. Under conditions with less metabolic stress, adipocytes could be able to handle the imbalance.

In summary, the present study demonstrates that adipocyte PIP5K1c plays an important role in regulating adipocyte expansion and glucose metabolism. PIP5K1c ablation results in impaired adipocyte expansion and ameliorates metabolic dysfunction under HFD challenge.

### Study strengths and limitations

The present animal study demonstrated that PIP5K1c was important for obesity. Deletion of PIP5K1c in adipocytes ameliorated obesity and insulin resistance, which is a potential target for the treatment of obesity. However, one limitation is that this study did not investigate the effect of deletion of PIP5K1c on mature adipocytes. Another limitation is that there is no evidence showing PIP5K1c expression between obese patients and healthy people. Further research will be conducted to establish this relationship.

## Conclusion

In conclusion, PIP5K1c regulates adipogenesis and adipose tissue homeostasis by regulating energy expenditure, especially under HFD feeding conditions. This study demonstrates a novel role of PIP5K1c in linking adipocyte signalling and glucose homeostasis. This could be a valuable alternative to control obesity and pathological metabolic syndrome.

## Supplementary Information

**Additional file 1.** Supplementary figures and tables.

## Data Availability

Data is available upon request.

## Abbreviations

NCD: normal chow diet
HFD: high-fat-diet
BMI: body mass index
BAT: brown adipose tissue
eWAT: epididymal white adipose tissue
subWAT: subcutaneous white adipose tissue
PIP5K1: type I phosphatidylinositol 4-phosphate 5-kinase
FA: focal adhesion
AUC: area under the curve
ITT: insulin tolerance test
GTT: glucose tolerance test
NEFA: nonesterified fatty acids
EE: energy expenditure
RER: respiratory exchange ratio
TG: triglyceride
TCH: total cholesterol
