# Supplementary material for: Adipocyte-specific deletion of PIP5K1c reduces diet-induced obesity and insulin resistance by increasing energy expenditure
Source: Lipids Health Dis. 2022 Jan 7;21:6. doi: 10.1186/s12944-021-01616-4 (PMC8742433; doi:10.1186/s12944-021-01616-4)
Supplement: Supplementary file 1 — Additional file 1. Supplementary figures and tables. [file 12944_2021_1616_MOESM1_ESM.docx]

**Adipocyte-specific deletion of PIP5K1c reduces diet-induced obesity and insulin resistance by increasing energy expenditure**

**
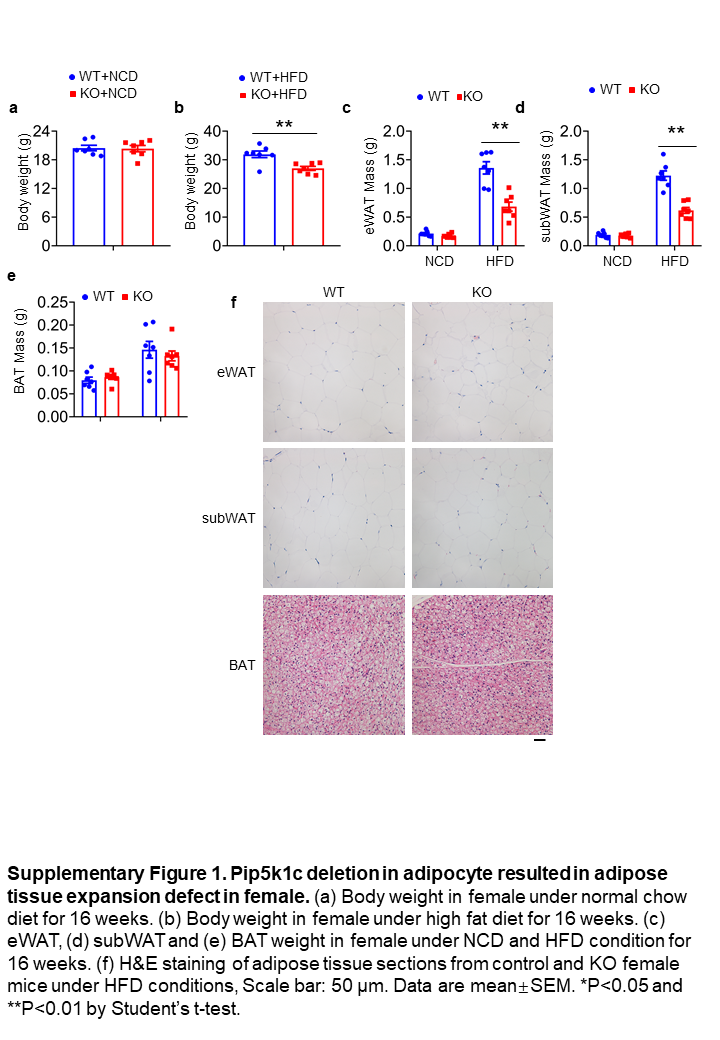
**

Supplementary Figure 1. Pip5k1c deletion in adipocyte resulted in adipose tissue expansion defect in female. (a) Body weight in female under normal chow diet for 16 weeks. (b) Body weight in female under high fat diet for 16 weeks. (c) eWAT, (d) subWAT and (e) BAT weight in female under NCD and HFD condition for 16 weeks. (f) H&E staining of adipose tissue sections from control and KO female mice under HFD conditions, Scale bar: 50 μm. Data are mean±SEM. *P<0.05 and **P<0.01 by Student’s t-test.


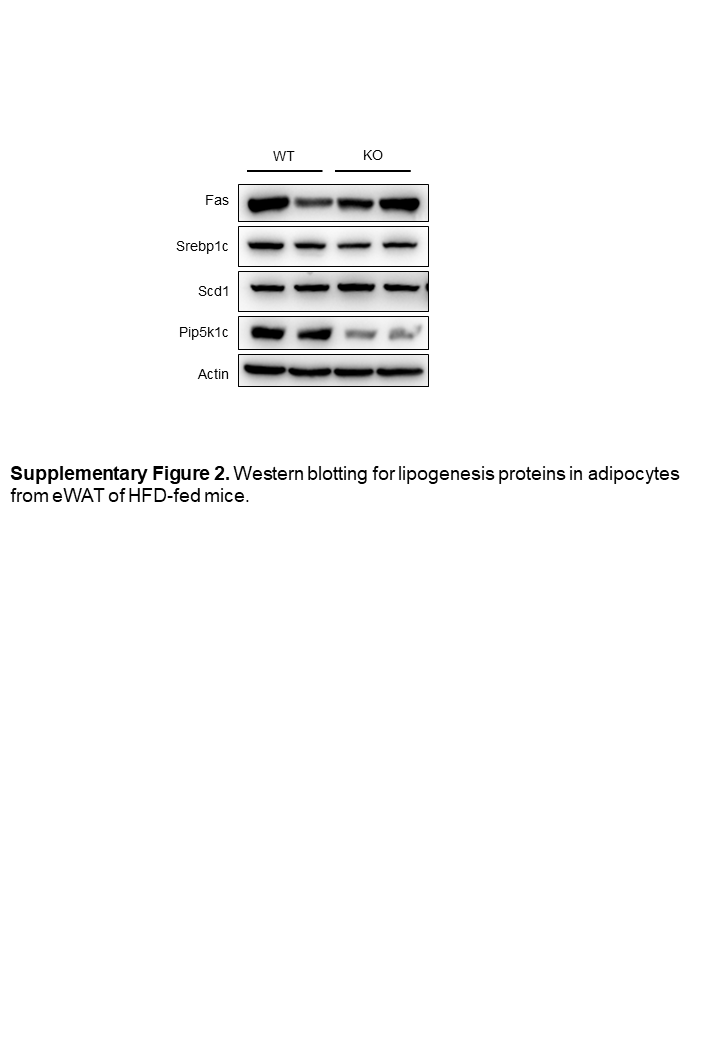


Supplementary Figure 2. Western blotting for lipogenesis proteins in adipocytes from eWAT of HFD-fed mice.

**
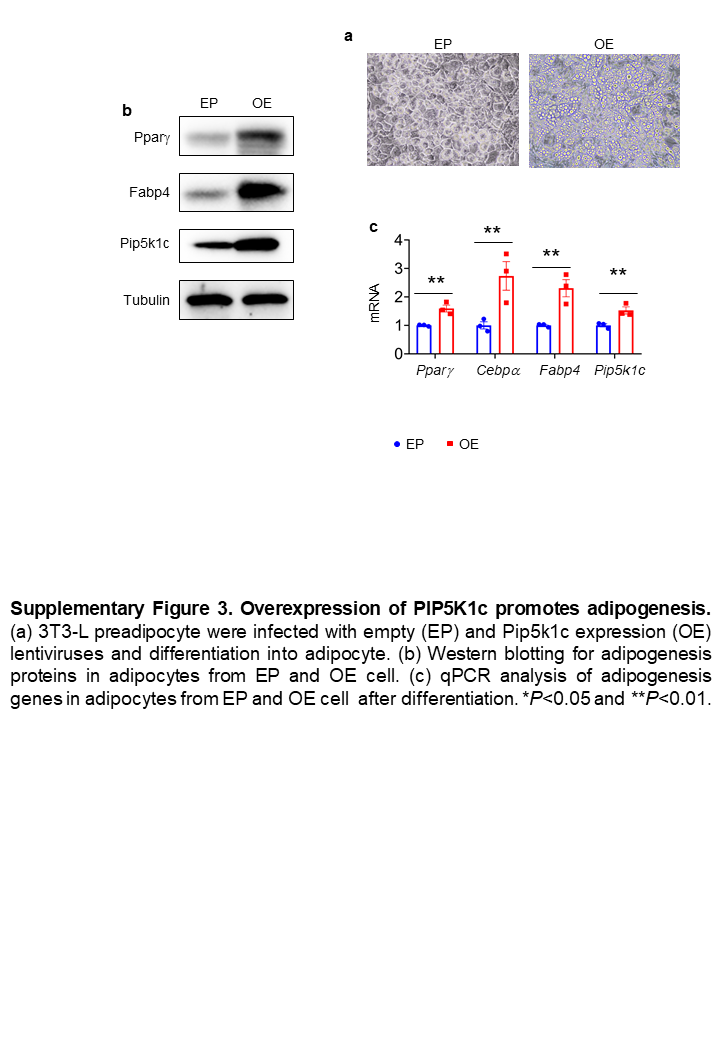
**

Supplementary Figure 3. Overexpression of PIP5K1c promotes adipogenesis. (a) 3T3-L preadipocyte were infected with empty (EP) and Pip5k1c expression (OE) lentiviruses and differentiation into adipocyte. (b) Western blotting for adipogenesis proteins in adipocytes from EP and OE cell. (c) qPCR analysis of adipogenesis genes in adipocytes from EP and OE cell after differentiation. **P*<0.05 and ***P*<0.01.

**Supplementary Table 1. Primer information for mouse**

| Gene | Forward | Reverse |
| --- | --- | --- |
| Fas | GCGATGAAGAGCATGGTTTAG | GGCTCAAGGGTTCCATGTT |
| Srebp1c | GGAGCCATGGATTGCACATT | GGCCCGGGAAGTCACTGT |
| Acc | GCGGCTACAGGGACTATACTG | CGGAAGTAAGAGCTACTAGCGG |
| Atgl | CCAAGGGGTGCGCTATGT | TTGGGTTGGTTCAGTAGGC |
| Hsl | TGCCCAGGATTGGATGGTTT | GTGAGAACGCTGAGGCTTTG |
| Pip5k1c | CTTGCAGAGGCCCCCTTG | GAGGTTGCTCCCTTCACTGG |
| Tnfa | CCACGTCGTAGCAAACCACC | GATAGCAAATCGGCTGACGG |
| Mcp1 | CACTCACCTGCTGCTACTCA | GCTTGGTGACAAAAACTACAGC |
| Il-6 | CTCATTCTGCTCTGGAGCCC | CAACTGGATGGAAGTCTCTTGC |
| Il10 | GGCGCTGTCATCGATTTCTC | ATGGCCTTGTAGACACCTTGG |
| Il1rn | AACGGAATGACAGCAGCACA | ATCCCAGATTCTGAAGGCTTGC |
| F4/80 | CACAGTACGATGTGGGGCTT | ACTGAGTTAGGACCACAAGGTG |
| Il-1b | TGCCACCTTTTGACAGTGATG | AAGGTCCACGGGAAAGACAC |
| Cd68 | ACTTCGGGCCATGTTTCTCTT | GGGGCTGGTAGGTTGATTGT |
| Cd36 | GCAGTGATTTGACTTGTGGC | TTTCAGAAGGCAGTACACAGAAG |
| Gapdh | TTTCTTCTTGCCTTGGGAGA | AGTTCCGCACTTCATTCAGG |
| Fabp4 | TGAAATCACCGCAGACGACA | ACACATTCCACCACCAGCTT |
| Cebpα | CCGTGGTGGTTTCTCCTTGA | TCATTTTTCTCTCACGGGGCCA |
| Pparγ | TTCGCTGATGCACTGCCTAT | GGAATGCGAGTGGTCTTCCA |
| Dgat1 | CAGACCAGCGTGGGCG | GAACAAAGAGTCTTGCAGACGATG |
| Acly | AAGAAGGAGGGGAAGCTGAT | TCGCATGTCTGGGTTGTTTA |
| Acot | CCCCGAGGTAAAAGGACCTG | TCTCAGGATAGTCACAGGGGG |

**Supplementary Table 2. Antibody information**

| **Name** | **Supplier** | **Cat no.** |
| --- | --- | --- |
| Pparγ | Cell Signaling technology | 2435s |
| Fabp4 | Cell Signaling technology | 2120s |
| Tubulin | Cell Signaling technology | 2128 |
| Pip5k1c | Cell Signaling technology | 3296s |
